# Mixture toxicity of chlorpyrifos-methyl, pirimiphos-methyl, and nonylphenol in Atlantic salmon (*Salmo salar*) hepatocytes

**DOI:** 10.1016/j.toxrep.2020.03.008

**Published:** 2020-04-06

**Authors:** Pål A. Olsvik, Liv Søfteland

**Affiliations:** a Nord University, Faculty of Biosciences and Aquaculture, Bodø, Norway; b Institute of Marine Research (IMR), Bergen, Norway

**Keywords:** Organophosphorus pesticides, Nonylphenol, Mixture toxicity, Shotgun lipidomics, Multivariate analysis, 3D cell culture

## Abstract

Pesticide formulations typically contain adjuvants added to enhance the performance of the active ingredient. Adjuvants may modify the bioavailability and toxicity of pesticides. In this study, the aim was to examine to which degree nonylphenol (NP) may interfere with the toxicity of two organophosphorus pesticides found in aquafeeds, chlorpyrifos-methyl (CPM) and pirimiphos-methyl (PPM). Atlantic salmon liver cells were exposed to these compounds singly or in combinations for 48 h using 3D cell cultures. Cytotoxicity, gene expression (RT-qPCR), and lipidomics endpoints were used to assess toxicity. The dose-response assessment showed that NP was the most toxic compound at equimolar concentrations (100 μM). Shotgun lipidomics pointed to a general pattern of elevated levels of saturated 18:0 fatty acids and declined levels of 18:1 monounsaturated fatty acids by the combined treatment. All three compounds had a distinct effect on membrane phospholipids, in particular on phosphatidylcholine (PC) and phosphatidylethanolamine (PE). Lipid species patterns predicted inhibited stearoyl CoA desaturase (SCD) activity and increased Δ6 desaturase (D6D) activity in co-treated cells. While all three compounds alone mitigated increased triacylglycerol (TAG) accumulation, combined treatment resulted in lower total TAG in the cells. Multivariate analysis with PLS regression showed significant combined effects for nine genes (*d5d*, *d6d*, *scd, srebf2*, *vtg*, *esr1*, *cyp1*, *ugt1a*, and *cat*) and four lipid species (FFA 22:5, LPC 18:0, TAG52:1-FA16:0, and TAG52:1-FA18:0). In summary, this study demonstrates that the adjuvant can be the main contributor to the toxicity of a mixture of two organophosphorus pesticides with relatively low toxicity in fish cells.

## Highlights

• Shotgun lipidomics points to combined effects on 18:0 and 18:1 lipid species.
• Combined effects seen on membrane phospholipids and TAG in salmon hepatocytes.
• Inhibited stearoyl CoA desaturase (SCD) and increased Δ6 desaturase (D6D) activity.
• Adjuvants may amend toxicity of active ingredient in pesticide formulations.

## 1 Introduction

Feed safety risk assessment is traditionally done on a contaminant-by-contaminant basis, potentially underestimating toxicity of chemical mixtures [1]. Mixture toxicity might be greater-than-additive (synergistic interaction) or less-than-additive (antagonistic interaction), and the outcome is often hard to predict. Dose addition is the most common way to examine the mixture toxicity of combinations of contaminants [1]. This concept is however not always valid when mixtures are composed of contaminants that exert their toxicity on different mechanisms, such as binding to different receptors. Mixture effects have been documented at concentrations below the experimental no observed adverse effect levels for single contaminants [2].

In recent years there has been increasing focus on the content of agricultural pesticides in aquaculture feeds. Limited availability of marine raw materials has forced the feed producers to increase the inclusion of plant ingredients in the products [3]. National monitoring programs have documented the presence of the organophosphorus pesticides chlorpyrifos-methyl (CPM) and pirimiphos-methyl (PPM) in 5–20 % of screened Norwegian Atlantic salmon feeds [4,5]. In 2018, the CPM and PPM concentrations ranged from 13 to 30 μg/kg and 9 to 19 μg/kg, respectively [5]. Vegetable oils are the most likely source of these pesticides in the commercial feeds. Organophosphorus pesticides act primarily by inhibiting acetylcholinesterase (AChE) in nerve cells [6]. Secondarily, mechanisms associated with oxidative stress, immunotoxicity, endocrine disruption, neurotoxicity, and neurobehavior may be affected [[7], [8], [9], [10]]. In addition to the active ingredient, pesticide formulations also contain adjuvants included to improve the solubility or the compatibility of the principal ingredient [11]. It is well documented that adjuvants may affect the bioavailability [11] and toxicity of pesticides [12,13]. Risk assessment of pesticide formulations should therefore also include adjuvants [14].

Internationally, nonylphenol ethoxylates (NPE) are among the most frequently used industrial surfactants [15,16]. NPEs are easily biodegraded, and some of their lipophilic metabolites such as nonylphenol (NP) are more persistent in the environment than the mother substance [17]. NPE metabolites have been detected in soybeans and maize products [18], as well as in fatty tissue of fish and other organisms [19]. Alkylphenols like nonylphenol (NP) are xenoestrogens and acts as endocrine disruptors [20], and can be very toxic to aquatic organisms [16]. The European Union has banned the production of NP and NPE due to their impacts on human health and the environment [15]. In the US, EPA is reviewing its use, while in Asian and South American countries NP is still widely available [16].

As part of seafood safety assessments, we have been studying the toxic effects of feed-borne contaminants in fish. In liver cells of Atlantic salmon, perturbation of lipid metabolism is one of the main secondary effect of organophosphorus pesticides such as chlorpyrifos (CPF), CPM and PPM. We have shown that exposure to these compounds leads to accumulation of lipids and disrupted cholesterol biosynthesis in liver cells [[21], [22], [23], [24], [25], [26], [27], [28]]. The mixture effects were mainly additive at low concentration and synergistic at higher concentration [21]. Furthermore, there is a lack of knowledge about how pesticide adjuvants affect the toxicity of CPM and PPM in fish. We selected NP as a model adjuvant with known toxic effects in fish. Despite being banned in the EU and being phased out as pesticide adjuvant and industrial surfactant, NP-based products are still in use in many countries. NP has been found in drainage water from agricultural areas and represent an environmental challenge in some countries from where the feed producers obtain their ingredients [29,30]. NP is a ubiquitous pollutant and may be present in fish feed independent of its use in pesticide formulations. We have detected NP above the detection limit in experimental control fish feed (unpublished data). It is therefore very relevant for mixture toxicity assessments of feed-borne contaminants. The aim of this study was therefore to perform a mixture toxicity evaluation of CPM, PPM and NP in Atlantic salmon liver cells.

## 2 Material and methods

### 2.1 *In vitro* exposure experiment

Hepatocytes were harvested with a two-step perfusion method from male Atlantic salmon (*Salmo salar*) (*n* = 6, mean ± st.dev.: 1.10 ± 0.32 kg). After cell extraction, cell viability was 95 ± 1% (Trypan Blue, *n* = 6, mean ± st.dev). Cells were cultured for 36−40 hours prior to exposure in L-15 medium, with change of medium after 18−20 hours. Hepatocytes used for lipidomics, gene expression (RT-qPCR) and the MTT assay were plated on laminin-coated (2 μg/cm^2^) 3D culture plates with Alvetex scaffolds (200 μm cross-linked polystyrene membranes, 42 μm mean void size, Reprocell, Glasgow, UK) or 96 2D xCELLigence plates. Cells were exposed in 12-well plates (lipidomics and for RT-qPCR), or in 96-well plates (cytotoxicity determination with MTT and xCELLigence). The following cell densities were used, 2.9 × 10^6^ cells per well for lipidomics and RT-qPCR, and 0.2 × 10^6^ cells per well for cytotoxicity screening. The exposure medium was changed after 18−20 hours. Cell harvesting and treatment are described in detail by Søfteland et al. [31]. The use of 3D culture plates with Alvetex scaffolds has been described in toxicity testing earlier by others [32,33].

Dose-response relationships were established for assessment of cytotoxicity and gene expression, using 0, 0.1, 1.0, 10, 100 and 1000 μM CPM, PPM and NP. For evaluation of mixture toxicity, the cells were exposed to the three chemicals using a factorial design with concentrations of 1, 50.5 and 100 μM as outlined in Table 1. CPM, PPM and NP were dissolved in dimethyl sulfoxide (DMSO) (Scientific and Chemical Supplies Ltd, Bilston, UK). All exposure solutions contained equimolar concentration of DMSO (0.2 %). The number of biological replicates was *n* = 5 for lipidomics and gene expression, and *n* = 6 for cytotoxicity screening. Chemicals were obtained from Sigma-Aldrich unless otherwise noted (Oslo, Norway).

**Table 1:** Factorial design applied to evaluate mixture toxicity.

The samples marked gray are part of the fractional factorial design used for the lipidomic analysis.

### 2.2 Cytotoxicity screening

Cell viability was assessed with the MTT tetrazolium assay according to the manufacturer’s protocol (*In Vitro* Toxicology assay kit, Sigma Aldrich). After the 48 -h treatment period, MTT substrate was added to the cell cultures. After 4 -h incubation, the number of viable cells was measured by recording changes in absorbance at 570 nm using a Labsystem iEMS microplate reader (Labsystems iEMS Reader MF, Helsinki, Finland). Cytotoxicity was also assessed with the impedance-based real-time xCELLigence system (Real-Time Cell Analyzer RTCA-SP, ACEA Biosciences, San Diego, USA) [34]. Cell index (CI) values and normalization was recorded using the RTCA Software. Real-time monitoring of cell viability was performed in an incubator at 10 °C without O_2_/CO_2_ supplementation with the RTCA single plate xCELLigence platform.

### 2.3 RNA isolation and RT-qPCR

Total RNA was extracted from hepatocytes cultured in Alvetex scaffold plates as described by the Reprocell protocol (Reprocell, Glasgow, UK) using the RNeasy Plus mini kit (Qiagen, Crawley, UK). In brief, cells were washed with PBS and lysed by adding 600 μL Qiagen RNeasy Plus mini kit lysis buffer RLT per well and placed for 10 min on a rotating platform (100 rpm) at room temperature. The lysate was homogenized 10 times with a 20-gauge needle. 600 μL 70 % ethanol was added to the homogenized lysate. A pipette was used to mix the sample 10 times before transfer to a collection tube and stored at −80 °C. The samples were transferred to RNeasy® spin columns. A DNase digestion on-column was performed before finalizing the RNeasy Plus mini kit protocol. RNA was eluted in 30 μL RNase-free MilliQ H_2_O and stored at −80 °C. The NanoDrop ND-1000 UV–vis Spectrophotometer (NanoDrop Technologies, Wilmington, DE, USA) and the Agilent 2100 Bioanalyzer (Agilent Technologies, Palo Alto, CA, USA) were used to check RNA quality. RNA integrity was evaluated with the RNA 6000 Nano LabChip kit (Agilent Technologies, Palo Alto, CA, USA). The mean RNA integrity number (RIN) of 12 randomly selected samples used for RT-qPCR was 10.0 ± 0.0 (mean ± st.dev.).

A two-step real-time RT-qPCR protocol was used to quantify the transcriptional levels of 11 target genes and potential 3 reference genes (see Table S1 for PCR assays). A normalization factor based upon *actb* and *eef1a1* (*M* < 0.42) was used to calculate mean normalized expression (MNE) of the target genes [35].

### 2.4 Lipidomics profiling

Harvesting of cells cultured in Alvetex scaffolds for the metabolomics analysis were performed according to the Reprocell protocol (Reprocell, Glasgow, UK). In brief, 3D hepatocytes were washed with PBS. The hepatocytes were incubated with 1.5 mL 0.25 % trypsin for 15 min on a rotating platform (100 rpm) at room temperature. 2 mL complete medium was used to stop the trypsin reaction. The cell suspension was transferred to centrifugation tubes and centrifuged at 1000×*g* for 5 min at 4 °C. The supernatant was discharged, and the cells were dissolved in 2 mL PBS and wasted a second time at 1000 × g for 5 min at 4 °C. Cells from three 12-well plate wells were pooled. Cells and medium were flash frozen and stored at −80 °C.

The lipidomics analysis was conducted by Metabolon (Metabolon, Research Triangle Park, NC, US) as earlier described by Zhang et al. [36]. In brief, lipids were extracted from samples using a modified Bligh-Dyer extraction in the presence of internal standards. Infusion-MS analyses were performed with a SelexION equipped Sciex 5500 QTRAP using both positive and negative mode electrospray. Each sample was analyzed twice, with IMS-MS conditions optimized for lipid classes monitored in each analysis. The 5500 QTRAP was operated in MRM mode to monitor the transitions for over 1100 lipids from up to 14 lipid classes. Individual lipid species were quantified based on the ratio of signal intensity for target compounds to the signal intensity for an assigned internal standard of known concentration. Lipid class concentrations were calculated from the sum of all molecular species within a class, and fatty acid compositions were determined by calculating the proportion of individual fatty acids within each class.

### 2.5 Statistics

One-way and two-way ANOVA with Holm-Sidak’s posttest were used for statistical analyses of the cytotoxicity and gene expression data (GraphPad Software, San Diego, CA, USA). The Surveyor software was used to analyze the lipidomics data using complex lipid panels (Metabolon, Research Triangle Park, NC, USA). To meet the assumption of homogeneity of variance, the data were log-transformed if deemed necessary before ANOVA analysis. For the lipidomics data, log-transformed differential composition values were used in all calculations. Principal component analysis (PCA) and PLS regression were used for multivariate analyses. Design of the experiment and PLS analysis was conducted with the MODDE software (Umetrics, Umeå, Sweden). Before PLS analysis the blend matrix was strengthened with interaction terms for the cytotoxicity and RT-qPCR data, but not for the lipidomics data for which a fractional factorial design was used. The PLS data were scaled to unit variance and mean centered. Finally, PLS model validation was ensured with respect to the explained variance and goodness of prediction (Q^2^), and with respect to goodness of fit (R^2^) [37].

## 3 Results

### 3.1 Nonylphenol acted cytotoxic

**Fig. 1:**
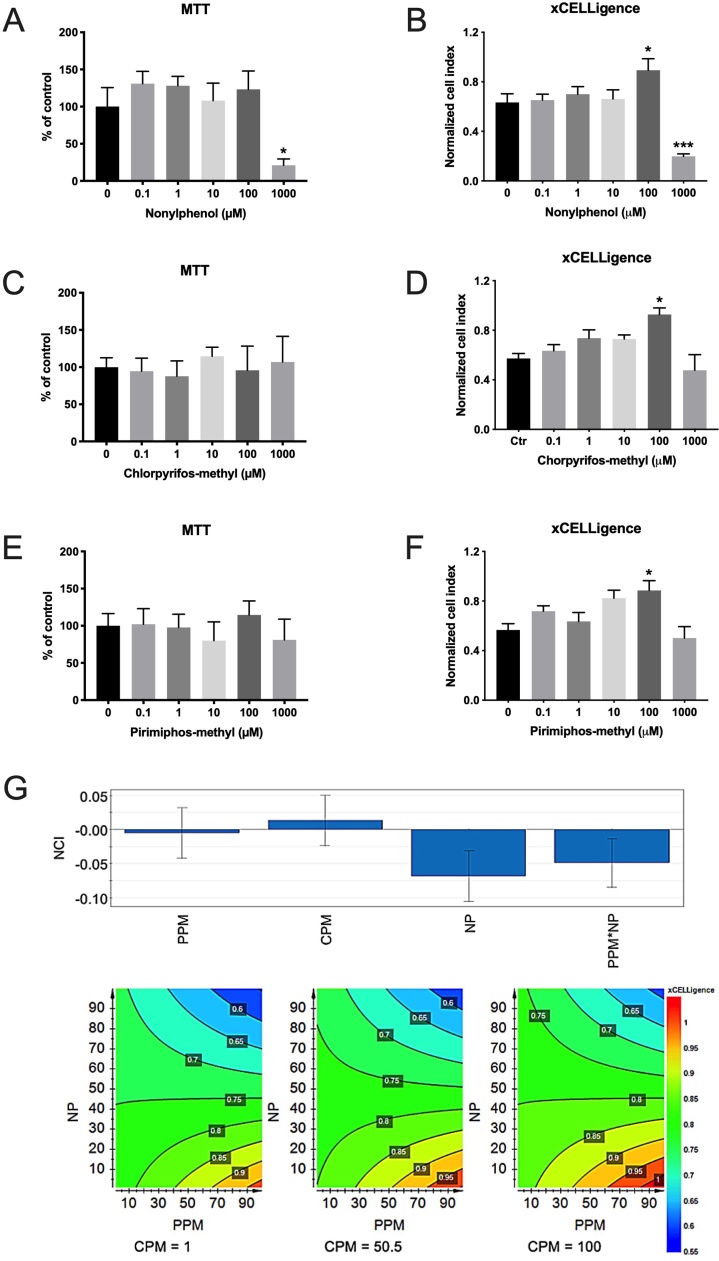
A-F) Dose-response-dependent cytotoxicity of chlorpyrifos-methyl (CPM), pirimiphos-methyl (PPM) and nonylphenol (NP) in Atlantic salmon hepatocytes determined with the MTT assay and the xCELLigence system. G) Simplified scaled and centered PLS regression coefficients with 95 % confidence intervals (upper figure) and contour plots (lower figure) based on normalized cell index (NCI) cytotoxicity (factorial design of xCELLigence data). The model contained one PLS component (R^2^ = 0.31 and Q^2^ = 0.10). The highlighted values in the contour plots represent NCI levels for the different stratification beddings (isoboles). The model was significant (regression: *p* = 0.001), with significant coefficients for NP (*p* = 0.0006) and PPM*NP (*p* = 0.0078) and showed a synergistic interaction between PPM and NP. Note, for the contour plots, blue color reflects the strongest effect with strongest reduction in NCI. (For interpretation of the references to colour in this figure legend, the reader is referred to the web version of this article).

Of the three examined compounds, only NP significantly reduced cell viability at the tested concentration range (Fig. 1). According to the MTT cytotoxicity data, 1000 μM NP reduced viability with 79 % (Fig. 1A), while the xCELLigence system, which measure impedance and how tightly the cells are bound to the gold-plated wells, showed a 69 % reduction in viability at 1000 μM NP (Fig. 1B). No cytotoxicity was seen up to 1000 μM for CPM and PPM (Fig. 1C–F), or for 100 μM of any of the compounds (the concentration used for the mixture toxicity assessment). The xCELLigence data indicated a stimulation effect at 100 μM for cells exposed to all three compounds, *i.e.* an improvement of their ability to bind to the substrate in the 96-well exposure plates. A multivariate PLS model, based on 54 log-transformed xCELLigence normalized cell index (NCI) values (9 mixture combinations and 6 fish, see Table 1), was applied to analyze the cytotoxicity data. The PLS model indicated a synergistic interaction on the cell viability reduction caused by PPM and NP at high concentrations (Fig. 1G).

### 3.2 Transcriptional effects on lipid metabolism, estrogenic, and detoxification markers

#### 3.2.1 NP gave the strongest dose-response effects

**Fig. 2:**
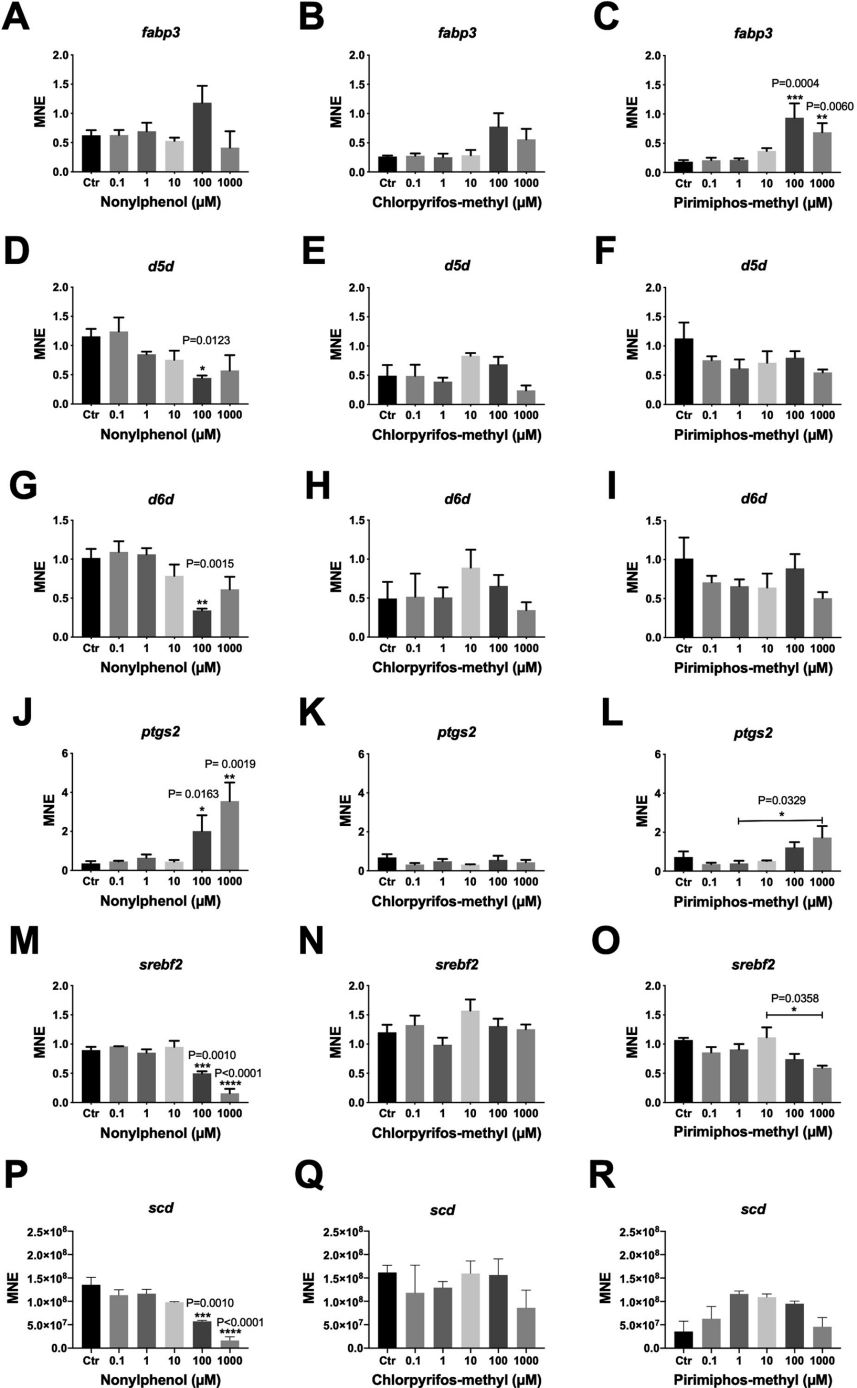
Dose-response relationships of genes associated with lipid metabolism in Atlantic salmon hepatocytes exposed to nonylphenol, chlorpyrifos-methyl and pirimiphos-methyl for 48 h. A-C) *fabp3*, D-F) *d5d*, G-I) *d6d*, J-L) *ptgs2*, M-O) *srebf2*, and P-R) *scd*. MNE = mean normalized expression. Asterisk’s denote significance based on one-way ANOVA analyses, with Holm Sidak’s posthoc test. *N* = 3–5. * *p* < 0.05 (exact p-values given in figure), ** *p* < 0.01, *** *p* < 0.001, **** p < 0.0001. Mean ± SEM.

Based on known markers for lipid metabolism (*fabp3*, *d5d (fads1)*, *d6d (fads2)*, *ptgs2*, *srebf2, scd*), endocrine disruption (*vtg*, *esr1*, *zp3*), oxidative stress (*cat*), growth and apoptosis (*igf1*), and detoxification (*cyp1a*, *cyp3a*, *gsta1*, *ugt1a*, *sult3*), selected genes were examined with RT-qPCR. For the lipid metabolism markers (Fig. 2), PPM at 100 and 1000 μM significantly up-regulated *fabp3* (Fig. 2C). Of the two delta desaturases, *d5d* and *d6d* were significantly down-regulated in cells exposed to 100 μM NP (Fig. 2D–G). NP further significantly up-regulated *ptgs2* at 100 and 1000 μM (Fig. 2J). Finally, *srebf2* and *scd* were significantly down-regulated at the two highest exposure concentrations by NP (Fig. 2M–P), while the other two compounds had no effects on their expression.

Of the estrogenic markers, *vtg* was significantly induced by NP (>1 μM), PPM (>10 μM) and CPM (>100 μM) (Supplementary file 1, Fig. S1). For NP and PPM, *vtg* showed the strongest induction at intermediate exposure concentrations (NP: 10 μM, PPM: 100 μM) (Fig. S1A–C). Compared to the control, *esr1* was significantly down-regulated by NP at 1000 μM, and significantly up-regulated by PPM at concentrations above 10 μM (Fig. S1D–F). The marker for growth and apoptosis, *igf1*, responded significantly only to NP treatment (up-regulated by 1000 μM NP) (Fig. S1J–L). *Cat*, included as a marker of oxidative stress, only showed a significant response in cells exposed to the highest NP dose (Fig. S1M–O). For the detoxification markers (Supplementary file 1, Fig. S2), significant responses were seen for *cyp1a* in cells exposed to PPM (up-regulated by 10 and 100 μM) (Fig. S2C), for *cyp3a* in cells exposed to NP (down-regulated by 100 and 1000 μM) (Fig. S2D) and for *ugt1a* in cells exposed to NP (up-regulated by 1000 μM) (Fig. S2 J).

#### 3.2.2 Interaction effects for eight transcripts

**Fig. 3:**
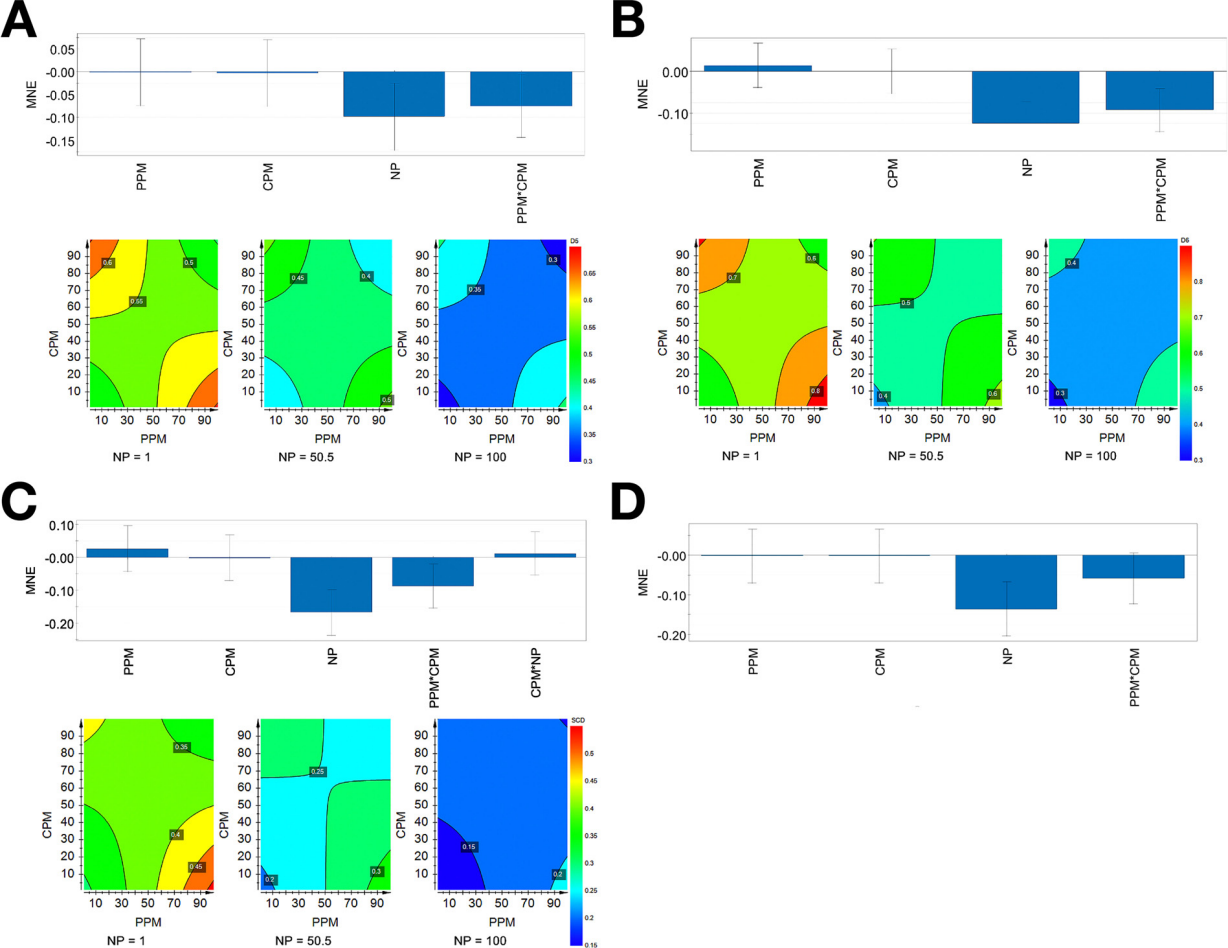
PLS models for lipid marker genes in chlorpyrifos-methyl (CPM), pirimiphos-methyl (PPM) and nonylphenol (NP) exposed Atlantic salmon hepatocytes. Simplified scaled and centered PLS regression coefficients with 95 % confidence intervals (upper figure) and contour plots (lower figure) based on mean normalized expression (MNE) (factorial design of RT-qPCR data). The model contained one PLS component. The highlighted values in the contour plots represent MNE levels for the different stratification beddings (isoboles). A) *d5d*, regression: *p* = 0.030, R^2^ = 0.23 and Q^2^ = 0.09, with significant coefficients for NP (*p* = 0.0100) and PPM*CPM (*p* = 0.036) and a synergistic and antagonistic interaction between PPM and CMP. B) *d6d*, regression: *p* = 0.000, R^2^ = 0.47 and Q^2^ = 0.22, with significant coefficients for NP (*p* = 0.0000) and PPM*CPM (*p* = 0.0007) and antagonistic interaction between PPM and CMP. C) *scd*, regression: *p* = 0.000, R^2^ = 0.45 and Q^2^ = 0.2, with significant coefficients for NP (*p* = 0.0002) and PPM*CPM (*p* = 0.011) and antagonistic and synergistic interaction between PPM and CMP. D) *srebf2*, regression: *p* = 0.003, R^2^ = 0.33 and Q^2^ = 0.2, with significant coefficients for NP (*p* = 0.0002) and no combined effect.

Multivariate PLS interaction assessment was performed using a factorial design and the transcriptional levels of 13 candidate biomarkers (*fabp3*, *d5d*, *d6d*, *ptgs2*, *scd*, *srebf2, vtg*, *esr1*, *cat*, *igf1*, *cyp1a*, *cyp3a*, *ugt1a*). The three genes which showed no significant dose-response effects, *gsta1*, *sult3* and *zp3*, were not subjected to multivariate analysis. Using MNE data, PLS regression models based on 45 log-transformed values (9 mixture combinations and 5 fish) were used to search for interaction effects between the three compounds at 1, 50.5 and 100 μM. According to the PLS models, significant effects were observed for 8 of the 13 target genes (Table S2). In all PLS models, NP had the strongest effect on the transcriptional levels compared to the other treatments. Significant interaction effects between CPM and PPM were observed for three lipid metabolism genes (*d5d*, *d6d* and *scd*). The contour plots indicated antagonistic interactions at low concentrations and synergistic interactions at high concentrations of the pesticides with downregulation of *d5d* (Fig. 3A) *d6d* (Fig. 3B) and *scd* (Fig. 3C), which turned only antagonistic for *d6d* and *scd*, with increasing NP concentration. No significant interaction terms were seen for *srebf2* (Fig. 3D).

The PLS models for two estrogenic markers and two detoxification markers were also significant (Fig. S3). For *esr1*, the PLS model indicated an antagonistic effect between PPM and CPM at all NP concentrations (Fig. S3A), while for *vtg* the model indicated both antagonisms and synergism over the NP concentration gradient (Fig. S3B). No significant combined effects were seen for *cyp1a* (Fig. S3C), while for *ugt1a* the model indicated a synergism and antagonism effect (Fig. S3D). The interaction effect for *cat* indicated a synergism and antagonism effect with increasing NP concentration (Fig. S3E). The synergistic and the antagonistic interaction on the down-regulation of these markers, caused by the pesticides, had an equal contribution to the mixture toxicity as the negative impact of NP.

### 3.3 Phospholipids and triacylglycerols (TAGs) distinctly affected by CPM, PPM and NP

**Fig. 4:**
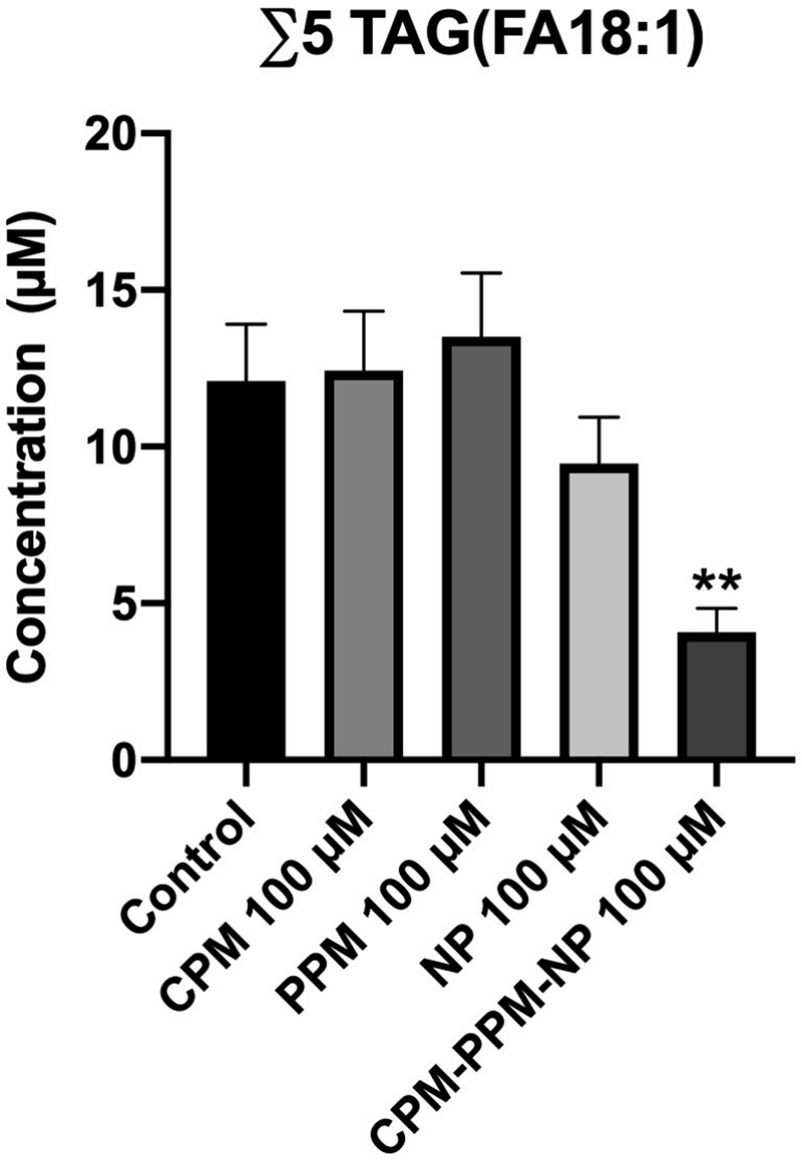
Total concentration of five TAG(FA18:1) lipid species (TAG50:2-FA18:1, TAG52:2-FA18:1, TAG52:3-FA18:1, TAG54:3-FA18:1 and TAG56:4-FA18:1) in Atlantic salmon hepatocytes exposed to 100 μM chlorpyrifos-methyl (CPM), 100 μM pirimiphos-methyl (PPM), 100 μM nonylphenol (NP) and a combination of 100 μM of all three compounds. *N* = 5. Mean ± SEM. **p < 0.01.

The number of lipid species detected in the hepatocyte culture samples used for dose-response assessment and fractional factorial design varied between 676 and 925 (*n* = 60, 870 ± 78 mean ± st.dev.). In total, the platform could quantify the concentration of 928 lipid metabolites. Total concentrations of each lipid class and mean difference relationships are shown in Supplementary file 2. The mean difference figures show significant lipid species separated by lipid class based on pairwise comparisons to the control for 1 and 100 μM CPM, PPM and NP, as well as for the cells treated with 100 μM of all three compounds. Table 2 shows a summary of the main effects on the different lipid classes. In brief, the three compounds had a distinct effect on a few free fatty acids and strongly affected phospholipids and TAG. While all three compounds reduced the total levels of phosphatidylethanolamine (PE) in the cells, exposure to CPM and NP increased the total levels of phosphatidylcholine (PC), whereas PPM resulted in decreased PC levels. Most surprisingly, although exposure to all three compounds alone (100 μM) resulted in accumulation of total TAG in the cells, combined exposure to 100 μM of all three compounds resulted in reduced total TAG level. Total TAG concentration dropped from 96.2 μM in the control to 63.7 μM in the co-treatment group (*p* = 0.042). This drop is illustrated for the five most strongly down-regulated TAG-FA18:1 species by the combined treatment in Fig. 4. For ∑TAG accumulation, 2-way ANOVA analysis showed a significant interaction effect between 100 μM PPM and the combined treatment groups (*p* < 0.0001), suggesting that PPM interfered with the degree of TAG accumulation in cells given a combination of all three compounds. TAGs bound to the saturated fatty acids palmitic acid (TAG-FA16:0) and oleic acid (TAG-FA18:0n-9), the monounsaturated fatty acids palmitoleic acid (TAG-FA16:1n-7), octadecaenoic acid (TAG-FA18:1n-9), and eicosenoic acid (TAG-FA20:1n-9), and the polyunsaturated fatty acids (PUFAs) linoleic acid (TAG-FA18:2n-6), eicosapentaenoic acid (EPA) (TAG-FA20:5n-3) and docosahexaenoic acid (DHA) (TAG-FA22:6n-3) were strongest affected by the studied compounds. Reduced levels of TAG were mirrored by reduced levels of diacylglycerol (DAG), a response most strongly driven by NP exposure. Table 3 shows the most significantly affected lipid species in cells treated with 100 μM of the three compounds. In terms of fold-change, NP had the strongest impact on the lipids.

**Table 2:** Summary of lipid effects. Chlorpyrifos-methyl (CPM), pirimiphos-methyl (PPM) and nonylphenol (NP). Neutral lipids: FFA = free fatty acid, DAG = diacylglycerol, TAG = triacylglycerol. Phospholipids: PC = phosphatidylcholine, PE = phosphatidylethanolamine, LPC = lysophosphatidylcholine. ↑ = increased 1-10-fold, ↑↑ = increased > 10-fold, ↓ = decreased 1-10-fold, ↓↓ = decreased > 10-fold.

Treatment	1 μM	100 μM
**CPM**	FFA(16:0) ↑	FFA(18:1) ↓
	PC(16:0/22:6) ↑	∑PC ↑
	∑TAG ↑	∑PE ↓
		∑TAG ↑↑
**PPM**	∑PC ↓	∑PC ↓
	∑PE ↓	∑PE ↓
	∑TAG ↓	∑TAG ↑↑
**NP**	∑PC ↑	∑DAG ↓
	∑PE ↓	∑PC ↑↑
	∑TAG ↓	∑PE ↓↓
		∑TAG ↑
**CPM + PPM + NP**		∑DAG ↓
		FFA(18:0) ↑
		∑LPC ↑
		∑PC ↓
		∑PE ↓↓
		∑TAG ↓↓

**Table 3:** The most significantly changed lipid species in Atlantic salmon liver cells treated with 100 μM chlorpyrifos-methyl (CPM), pirimiphos- methyl (PPM) and nonylphenol (NP).

Treatment (100 μM)	Up-regulated	Fold-change	P-value	Down-regulated	Fold-change	P-value
**CPM**	TAG58:6-FA22:5	2.36	<0.001	CE(24:1)	−2.46	0.018
	TAG56:5-FA22:5	2.26	0.008	PE(P-18:0/18:0)	−2.28	<0.001
	TAG56:8-FA20:5	2.24	0.023	FFA(22:6)	−2.00	0.022
	TAG56:9-FA20:5	2.18	0.042	PI(16:0/20:3)	−1.82	0.022
	TAG52:7-FA20:5	2.15	0.004	PE(18:1/18:2)	−1.78	0.003
**PPM**	TAG58:7-FA18:0	2.26	<0.001	CE(24:1)	−2.13	0.031
	TAG58:7-FA22:6	2.17	<0.001	PE(16:0/20:3)	−1.27	0.029
	TAG52:7-FA22:6	2.09	0.003	PC(18:1/20:3)	−1.25	0.017
	TAG58:6-FA18:0	2.04	<0.001			
	TAG58:6-FA22:5	2.04	0.003			
**NP**	TAG58:6-FA18:0	5.12	<0.001	PI(18:1/18:1)	−2.78	0.003
	TAG56:5-FA22:5	5.03	<0.001	PC(12:0/20:3)	−2.45	0.007
	TAG56:6-FA22:6	5.00	<0.001	CE(24:1)	−2.43	0.015
	TAG54:0-FA18:0	3.98	0.023	PE(18:1/20:4)	−2.30	<0.001
	TAG58:7-FA18:0	3.80	<0.001	PE(18:1/22:5)	−2.26	0.001
**CPM + PPM + NP**	PI(18:1/18:2)	4.28	<0.001	PI(18:1/20:3)	−4.20	<0.001
	LPC(18:0)	3.83	<0.001	PI(18:1/20:4)	−3.96	<0.001
	PI(18:0/18:1)	3.08	0.004	TAG48:2−12:0	−2.81	0.035
	LPC(22:5)	2.64	0.008	PC(12:0/18:1)	−2.79	0.001
	LPC(17:0)	2.57	0.003	TAG48:2-FA14:1	−2.70	0.017

**Fig. 5:**
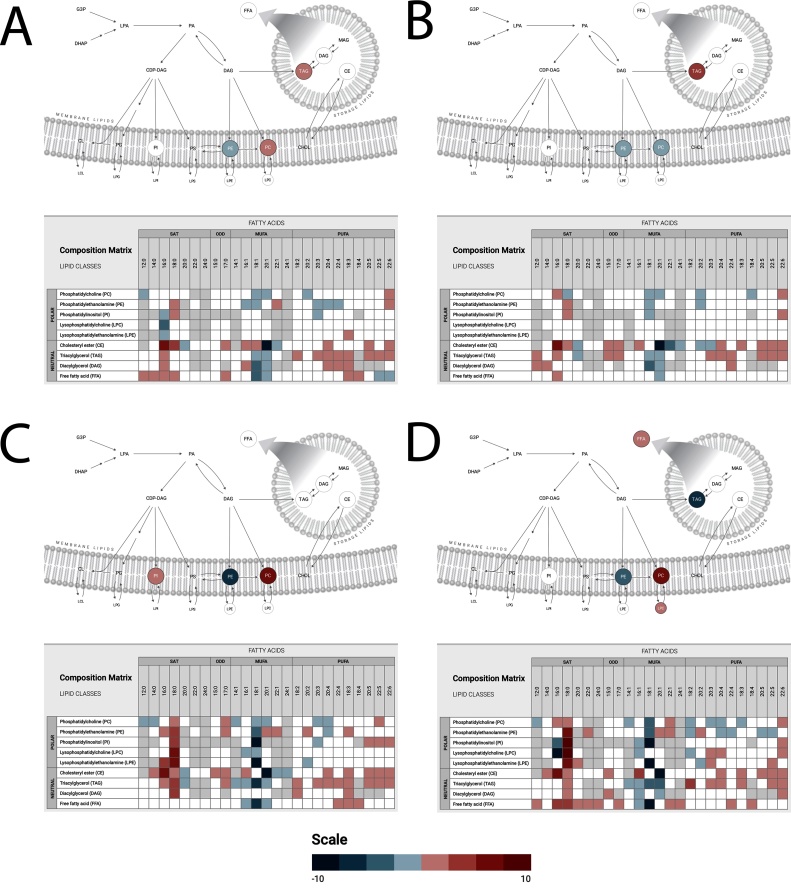
Complex lipid pathway maps in Atlantic salmon hepatocytes exposed to A) 100 μM chlorpyrifos-methyl, B) 100 μM pirimiphos-methyl, C) 100 μM nonylphenol and D) a combination of 100 μM of all three compounds (generated with Metabolon’s Surveyor tool). Significance levels are based on mole percent data, mean differences and log-transformed P-values (*p* < 0.05).

Fig. 5 shows complex lipid pathways and maps in Atlantic salmon hepatocytes exposed to 100 μM CPM (Fig. 5A), 100 μM PPM (Fig. 5B), 100 μM NP (Fig. 5C) and a combination of 100 μM of all three compounds (Fig. 5D). Among the most distinct effect of CPM, PPM and NP alone and in combination was the impact on saturated cholesteryl ester (elevated 16:0), and monounsaturated cholesteryl ester (reduced 20:1). NP alone also profoundly increased lysophosphatidylcholine (LPC) and lysophosphatidylethanolamine (LPE) (18:0), and reduced phosphatidylinositol (PI), PE and free fatty acid (FFA18:1). Combined treatment elevated the levels of 18:0-bearing species and declined the levels of 18:1 species. Phosphatidylinositol (PI), lysophosphatidylcholine (LPC), lysophosphatidylethanolamine (LPE), and PE were the most strongly increased saturated lipid species (18:1) (in that order), while phosphatidylinositol (PI), lysophosphatidylethanolamine (LPE), and free fatty acid were the most strongly reduced saturated lipid species.

**Fig. 6:**
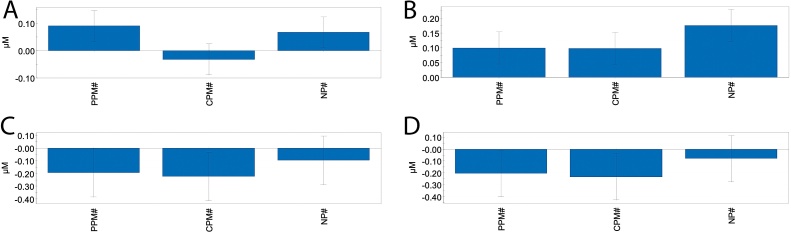
PLS models of four lipid species showing significant contribution in Atlantic salmon hepatocytes after exposure to chlorpyrifos-methyl (CPM), pirimiphos-methyl (PPM) and nonylphenol (NP). Simplified scaled and centered PLS regression coefficients with 95 % confidence intervals based on lipid levels (fractional factorial design). The model contained three PLS components. A) Free FA (22:5), regression: *p* = 0.0040, R^2^ = 0.55 and Q^2^ = 0.38, with significant coefficients for PPM (*p* = 0.0030) and NP (*p* = 0.022). B) LPC (18:0), regression: *p* = 0.0000, R^2^ = 0.83 and Q^2^ = 0.69, with significant coefficients for PMM (*p* = 0.0013), CPM (*p* = 0.0015), and NP (*p* = 398-006) C) TAG52:1-FA16:0, regression: *p* = 0.028, R^2^ = 0.42 and Q^2^ = 0.17, with significant coefficients for PPM (*p* = 0.0475), CPM (*p* = 0.026), and D) TAG52:1-FA18:0, regression: *p* = 0.025, R^2^ = 0.43 and Q^2^ = 0.19, with significant coefficients for PPM (*p* = 0.0403), CPM (*p* = 0.0216).

For lipid species, a fractional factorial design was used to search for associations between the three compounds. A total of 48 lipid species were selected for multivariate analysis. These included the 10 most significantly affected lipid species obtained from the dose-response relationship assessment (at 100 μM), as well as all free fatty acids. Of the 14 lipid species for which the PLS regression model was significant (Table S3), most changes were caused by one chemical except for four lipids where two or three of the chemical contributed to the observed responses; FFA (22:5) (Fig. 6A), LPC (18:0) (Fig. 6B), TAG52:1-FA 16:0 (Fig. 6C), and TAG52:1-FA 18:0 (Fig. 6D). Based on the linear terms, only LPC (18:0) was affected by all three chemicals co-acting to increase LPC levels.

## 4 Discussion

This study shows how an adjuvant with endocrine-disrupting properties may influence the toxicity of organophosphorus pesticides in fish cells. NP most distinctly affected the degree of total TAG accumulation in cells co-treated with CPM and PPM in Atlantic salmon hepatocytes. While NP, CPM and PPM (100 μM) alone all resulted in increased accumulation of ∑TAG, combined treatment had the opposite effect. Short-term exposure to CPF/CPM typically leads to accumulation of monoacylglyceride (MAG) and reduced levels of many PUFAs [21,23,26], whereas PPM increases the levels of diHOME fatty acids and cholesterol and decreases lysophospholipid metabolism in salmon hepatocytes [24]. In this study, using a lipidomics pipeline that did not analyze individual MAGs, combined treatment showed that PPM and CPM contributed to the reduced levels of palmitic acid-TAG (TAG 52:1-FA 16:0) and stearic acid-TAG (TAG 52:1-FA 18:0). However, only NP contributed to the reduction of TAG (50:1-FA 16:0) in co-exposed cells. Taken together, mixture toxicity assessments are of major importance to understand how these chemicals together affect accumulation of lipids in the cells.

Exposure to persistent organic pollutants (POPs) and especially endocrine disruptors is often associated with accumulation of fat in the liver [38]. Polyethoxylated tallow amines, adjuvants used in glyphosate-based herbicide formulations, and nonylphenol ethoyxlate surfactants, can all induce TAG accumulation in mice adipocytes [39]. Excessive accumulation of lipids in the liver has been suggested to be one of the most pathologically recognized responses to chemical exposure [40]. Fish studies indicate that mechanisms associated with excessive synthesis and accumulation of TAG are conserved across phyla [41,42]. In this study, we observed a synergistic interaction between NP and PPM on cell death, and that both NP and PPM reduced total TAG at 1 μM, suggesting that NP and PPM most profoundly affected lipid accumulation.

Combined treatment resulted in a general pattern of elevated levels of saturated fatty acids and a declined level of monounsaturated fatty acids. In particular, the increased levels of 18:0-bearing species across all classes and reduced levels of 18:1 species for most classes, was more pronounced in cells co-treated with all three compounds. Reduced levels of 18:1 species suggests an inhibition of stearoyl CoA desaturase (SCD), an enzyme responsible for endogenous biosynthesis of monounsaturated fatty acids, *i.e.* palmitoleic acid (16:1n-7) and oleic acid (18:1n-9), giving rise to a mixture of 16:1 and 18:1 unsaturated fatty acids [43]. In support, exposure to the mixture of all three compounds resulted in reduced transcriptional levels of *scd*. As palmitoleic acid and oleic acid are major components of membrane phospholipids, inhibited SCD activity may affect membrane permeability and fluidity. A distinct reduction of PE and increase of PC, the most common phospholipids in fish membranes [44], by CPM, NP and combined treatment emphasizes that membrane phospholipids are one of the main targets of the studied compounds and in particularly of NP. Long-term *in vivo* studies also point to effects of organophosphorus pesticides on phospholipids. We observed a significant reduction in arachidonic acid (ARA, 20:4n-6) and increased content of the saturated fatty acid palmitic acid (PA, 16:0) with increasing dietary content of CPM in liver of Atlantic salmon [28]. Up-regulation of *ptgs2*, of which the encoded protein COX2 converts ARA to prostaglandin H2 [45], indicates an inflammatory effect at 100 and 1000 μM NP, a response not seen for CPM and PPM, and an effect on membrane phospholipids in this study. The significant elevated levels contributed by PPM and NP seen for free fatty acid docosapentaenoic acid (DPA, 22:5) could be linked to differential expression of *ptgs2*, as DPA acid has been shown to affect the expression of PTGS2 protein in rat cells [46]. NP alone strongly increased PC (FA 16:0), PC (FA 18:0) and PC (FA 22:6), and reduced PE (FA 18:1) and PE (FA 22:6). PE, together with fatty acid desaturation and cholesterol, is a key regulator of membrane fluidity in most eukaryotic cells [47]. Altered levels of PC and PE could also be associated with increased incorporation of excess intracellular fatty acids into TAGs stored in cytosolic lipid droplets which are surrounded by a monolayer of phospholipids [48]. Furthermore, an impact on membrane fluidity was suggested by the reduced transcriptional levels of *srebf2* by NP and that in combined treatment only NP contributed to the down-regulation seen for this gene. *Srebf2*, encoding SREBP2, is a transcription factor that regulates the synthesis and cellular uptake of cholesterol and fatty acids, two major constituents of cell membranes [49]. Lysophosphatidylcholine (LPC 18:0) was the only lipid species that showed a significant increase that was caused by all three compounds in co-exposed cells. Lysophosphatidylcholine (LPC 18:0) is a membrane lysophospholipid found in small amounts in most tissues [50] that has a role in lipid signaling by acting on lysophospholipid receptors and it also acts as a membrane stabilizer. Interestingly, lysophosphatidylcholine (LPC 18:0) has an industrial application as a surfactant and emulsifier [50]. Based on the lipid signature equations, fatty acid synthase (FASN) activation was only predicted for the combined treatment. The function of FASN is mainly to catalyze the synthesis of palmitoleic acid from acetyl-CoA and malonyl-CoA into long-chain saturated fatty acids [51]. FASN activation again points to effects on mechanisms associated with the degree of unsaturation (number of double bonds) and membranes [52,53]. According to the lipidomics data, NP contributed most strongly to SCD inhibition, but CPM and PPM antagonized the reduction. While all three compounds reduced the total levels of PE in the cells, exposure to CPM and NP increased the total levels of PC, whereas PPM resulted in decreased PC levels. The transcriptomic data also suggested that this was mainly a response to NP exposure, as *scd* was down-regulated by 100 and 1000 μM NP but not by the two organophosphorus pesticides. In zebrafish, it had been shown that CPF upregulate *scd* [54], indicating that the studied compounds act differently on this gene. It is well established that cell membranes and phospholipids are among the main targets of agricultural adjuvants in animals [55,56]. In our earlier research, we have documented this to be true for CPM and PPM in Atlantic salmon hepatocytes [25,28]. In this study, we show that NP acts by disrupting phospholipid metabolism by reducing the levels of PE and increasing the levels of PC and phosphatidylinositol (PI). This effect was less pronounced in co-treated cells, again suggesting a different mechanistic effect of the studied compounds on phospholipids.

One of the novel lipidomics findings in this study was the predicted dysregulation of D6D activity in cells treated with a combination of all three compounds, a response not seen in separately treated cells. Additionally, significant interaction effects were observed for all of the desaturase genes (*d5d*, *d6d*, and *scd*). D6D, together with D5D, are key enzymes in endogenous production of long-chain PUFAs (n-3 and n-6 PUFAs) from monounsaturated fatty acids [57]. All three compounds alone resulted in reduced levels of very-long chain fatty acids, a response not seen in co-treated cells. At the transcriptional level, *d6d* (and *d5d*) was down-regulated by NP at 100 μM, but not at 1000 μM. Treatment with CPM and PPM did not significantly affect *d6d* transcription. We have previously seen a reduced transcription of *d6d* in Atlantic salmon liver cells exposed to 1000 μM PPM [24], a finding not replicated in the current study. We have generally noticed that 3D cell cultures are less responsive (*i.e.* better protected) than 2D cell cultures to external stimuli, possibly explaining the contradicting result. Dietary fat content as well as many POPs are known to modify *d6d* expression in fish. For example, transcriptional down-regulation has been reported in Javanese medaka (*Oryzias javanicus*) exposed to bisphenol A [58] and in zebrafish (*Danio rerio*) larvae exposed to 2,2',4,4'-tetrabromo diphenyl ether (PBDE) [59]. In an earlier study, we found a significant interaction effect between dietary lipid composition (high fish oil *versus* high plant oil content) and MeHg for *d5d* but not for *d6d* transcription in Atlantic salmon liver after prolonged exposure [60], further emphasizing an association between contaminant exposure and effects on desaturase genes. The contour plots in this study showed an antagonistic effect of PPM and CMP at low concentrations and synergistic effect at high pesticide concentrations on NP down-regulation of *d5d* and antagonistic effect on NP down-regulation of *d6d*, suggesting that potent estrogenic chemicals like NP at high concentrations may strengthen the toxic effect of feed-borne organophosphorus pesticides in salmon. D6D, encoded by *d6d_a/b/c* in Atlantic salmon [57], is also a rate-limiting enzyme involved in the conversion of plant-based alpha-linolenic acid (ALA, 18:3n-3) into longer chain n-3 PUFAs such as eicosapentaenoic acid (EPA, 20:5n-3) and docosahexaenoic acid (DHA, 22:6n-3) [61]. Detrimental effects on D6D activity by feed-borne pesticides may potentially be a challenge for the salmon industry, since this enzyme is responsible for the conversion of dietary alpha-linolenic acid into healthy PUFAs in salmonids. With the reduced inclusion of marine ingredients in feed, the levels of EPA and DHA in Scottish and Norwegian farmed salmon filet has dropped from an average of 2.7 g in 2006 to 1.4 g per 100 g fillet and 1.1 g per 100 g fillet in 2015 [62,63]. To keep the nutrition value of farmed salmon as high as possible, the salmon’s innate capacity to produce n-3 PUFAs from 18:3n-3 is of paramount importance for the aquaculture industry [61]. Follow-up *in vivo* experiments will be required to determine whether pesticide-induced impacts on D6D will affect fish health, and whether this will occur at concentrations found in present-day fish feed.

As expected, the three compounds induced dose-response effects on *vtg* and *esr1*, included in this study as transcriptomic markers for endocrine disruption in male fish [64]. For NP and PPM, the response curves were non-monotonic, with strongest *vtg* induction at 10 μM for NP and at 100 μM for PPM. In terms of estrogenicity, the range was NP > PPM > CPM. NP is a chemical with a well-documented endocrine-disrupting potential in fish [65]. The findings for CPM and PPM are in line with our previous *in vitro* studies, which have documented that these compounds have relatively weak estrogenic activity in Atlantic salmon liver cells [[22], [23], [24],^26^]. After short-term waterborne *in vivo* exposure, CPF has been shown to be able to induce VTG in male zebrafish liver [66]. After prolonged dietary exposure, however, we have not detected any sign of endocrine disruption by CPM in liver of Atlantic salmon [25] or in Atlantic cod (*Gadus morhua*) [27]. In mice, CPM is considerably less toxic than CPF [67], possibly explaining the difference. This study also document mixture toxicity for these genes between NP and the two organophosphorus pesticides. In Atlantic salmon liver cells exposed to a contaminant mixture consisting of CPF, it was shown that EPA had an antagonistic effect on *vtg* transcription [22]. Taken together, these results suggest that mixture toxicity should be considered when evaluating the toxic effect of adjuvants with estrogenic capacity in fish.

Based on both the transcriptomic and lipidomic examination, this study clearly shows that NP is a stronger toxicant than CPM and PPM at equimolar concentrations. In addition to the aforementioned genes, the dose-response curves seen for *igf1*, *cat*, *cyp3a* and *ugt1a* illustrate a more distinct response pattern for NP than for CPM and PPM. The increased expression of *igf1* by NP may be associated with elevated levels of *ptgs2*, as the latter has been shown to induce this cytokine [68]. However, overexpression of *igf1* has been linked to numerous phenotypes and pathways, including apoptosis, and could signal increased cell death by 1000 μM NP as indicated by the increased cytotoxicity in this group. Somewhat surprisingly, NP did not affect *cyp1a* transcription. Xenoestrogens like NP are known to suppress total microsomal CYP content due to inhibitory aryl hydrocarbon and estrogen receptor crosstalk [69]. NP did however down-regulate *cyp3a* at 100 and 1000 μM, and *ugt1a* was up-regulated by the highest NP concentration. We have earlier observed increased *cyp1a* transcription at intermediate CPF concentrations (10 μM) but not at higher concentrations (100 μM) after *in vitro* exposure [21,23], a response not replicated with the less toxic CPM in this study. Such inverted U-shape response curves for *cyp1a*, as also suggested by the significant up-regulation of PPM at intermediate concentrations in this study, has been documented earlier in zebrafish after CPF exposure [70] and is often reported for endocrine disrupting chemicals [71]. Receptor crosstalk and non-monotonic response curves illustrate the complexity of mixture exposure assessments [72]. In terms of detoxification mechanism, 3D cell cultures have been shown to offer a few advantages over using 2D culture. For example, we have seen expression of genes encoding drug transporters in Atlantic salmon 3D cultures, a phenomenon not experienced using 2D cell cultures (unpublished work). Similar results have been reported by others. Breslin and O’Driscoll [73] observed increased expression of a number of proteins involved in cell survival, drug targeting and drug transporters in 3D compared to 2D cultures using human breast cancer cell lines. Reduced cytotoxicity using 3D culture compared to 2D cell culture has also been reported for human monocytic leukemia cells [74], suggesting that 3D cultures have a more robust defense against external stressors.

The dose-response curve for *cat* suggests that NP induced oxidative stress only at 1000 μM, while no significant effects were seen for the two organophosphorus pesticides. We have earlier documented that CPF and CPM induce oxidative stress in Atlantic salmon hepatocytes at 100 μM based on global metabolomics data, but not according to changed gene expression (RNA-seq) [23,26], while PPM appears to be a weak oxidant [24]. With significant interaction PLS models seen for *ugt1a* and *cat*, this study clearly suggest that mixture toxicity should be considered also for mechanisms associated with detoxification and oxidative stress when studying organophosphorus pesticides and adjuvants with estrogenic potential.

In line with earlier findings [14], this study underlines the importance of considering mixture toxicity when examining the potential harmful effect of single pesticides. The main challenge for mixture toxicity evaluations is to predict the effects of a combination of chemicals based only on information for each compound individually, including toxicological mechanism of action [75]. As we have documented earlier for CPM and PPM, these organophosphorus pesticides mainly act antagonistic at low concentrations and synergistic at higher concentrations. This finding is in agreement with earlier research showing that cholinesterase inhibitors often act synergistically [76]. Furthermore, our study indicates that the pesticides, when given in high concentration, act synergistically especially on secondary stress markers in the cells. This is notably distinct for the oxidative stress marker *cat*. Interestingly, at 100 μM NP the contour plots for the two estrogenic markers *vtg* and *esr1* suggest an antagonistic effect at low CPM and PPM concentrations. The down-regulation of these transcripts was severely strengthened by NP (additive effect). This probably reflects that NP is a much stronger xenoestrogen than the two organophosphorus pesticides. For the lipids which showed significant combined effects, the three chemicals were driving the response in the same direction for three lipid species, while for FFA(22:5) the data suggested that only PPM and NP were responsible for the increased FFA(22:5) levels observed in mixture exposed cells, whereas CPM acted differentially. Except for the total level of PE, and TAG accumulation after combined exposure, our data suggest that these compounds disrupt many mechanisms associated with lipid metabolism in a similar way. Follow-up *in vivo* studies should also use lower concentrations, as recent findings indicate that long-term exposure to chemical mixtures of contaminants with similar toxic action may induce adverse effects in animals even at doses below their toxicological reference value [77,78]. Overall, this study shows that even with compounds that only have partly overlapping mode of action, mixture toxicity may lead to underestimation of the negative health effects in risk assessment of feed-borne pesticides in farmed fish.

## Author contributions

LS conceived and designed the experiment, and conducted the experimental work. PO and LS analyzed and interpreted the transcriptomic and lipidomics data. PO wrote the manuscript with support from LS. Both authors critically revised and approved the final manuscript.

## Declaration of Competing Interest

The authors declare that they have no known competing financial interests or personal relationships that could have appeared to influence the work reported in this paper.
