# Supplementary material for: Mixture toxicity of chlorpyrifos-methyl, pirimiphos-methyl, and nonylphenol in Atlantic salmon (Salmo salar) hepatocytes
Source: Toxicol Rep. 2020 Apr 6;7:547–58. doi: 10.1016/j.toxrep.2020.03.008 (PMC7191540; doi:10.1016/j.toxrep.2020.03.008)
Supplement: Supplementary file 1 [file mmc1.docx]

**Table S1.** PCR assays.

|  |  |  |  |  |  |  |  |
| --- | --- | --- | --- | --- | --- | --- | --- |
|  |  |  |  |  |  |  |  |
| **Gene symbol** | **Gene name** | **Potential marker for** | **Accession no.** | **Forward primer** | **Reverse primer** | **Amplicon size (bp)** | **PCR efficiency** |
|  |  |  |  |  |  |  |  |
|  |  |  |  |  |  |  |  |
| *cyp1a* | Cytochrome P450 family 1 subfamily A | Detoxification | NM_001123687 | ATCGGACGCAACGAGGTCTA | TGACAGCGCTTGTGCTTCAT | 128 | 2.02 |
| *cyp3a* | Cytochrome P450 family 3 subfamily A | Detoxification | DQ361036 | ACTAGAGAGGGTCGCCAAGA | TACTGAACCGCTCTGGTTTG | 146 | 1.98 |
| *gsta1* | Glutathione S-transferase alpha 1-like | Detoxification | NM_001141492 | CTGGCCACGGAGATCAAACT | GGAAGGCGTAGGCAATCACA | 124 | 1.99 |
| *ugt1a* | UDP glucuronosyltransferase family 1A-like | Detoxification | DY802180 | ATAAGGACCGTCCCATCGAG | ATCCAGTTGAGGTCGTGAGC | 113 | 2.01 |
| *sult3* | Sulfotransferase family 3-like | Detoxification | BT049255 | CTGGACAGGCTGTGCTCCTT | GTAGTTGCCATGCTGTTCTTCT | 111 | 1.98 |
| *vtg* | Vitellogenin | Endocrine disruption | DY802177 | AAGCCACCTCCAATGTCATC | TCTCACTAAACGGAGCAGGAT | 128 | 2.02 |
| *esr1* | Estrogen receptor 1 | Endocrine disruption | >Contig15966_Atlantic_salmon | GGTCTCCCCAGCCAGTCATA | TGGAGGTGATGCAGAGCTTCT | 112 | 2.06 |
| *zp3* | Zona pellucida glycoprotein 3 | Endocrine disruption | CK991165 | CTGCGGCTGAATTGGGTTAC | ACTCCCAGTTCATGCCTCGTT | 101 | 1.99 |
| *igf1* | Insulin-like growth factor 1 | Growth | M81904 | TGACTTCGGCGGCAACA | GCCATAGCCCGTTGGTTTACT | 119 | 1.97 |
| *fabp3* | Fatty acid binding protein 3-like | Lipid metabolism | BT125322 | CCGCCGACGACAGAAAAA | TTTTGCACAAGGTTGCCATTT | 61 | 2.03 |
| *d5d* | Delta 5-desaturase / Fatty acid desaturase 1 (fads1) | Lipid metabolism | NM_001123542 | GGAACCACAAACTGCACAAGT | GTGCTGGAAGTGACGATGGT | 83 | 1.92 |
| *d6d* | Delta 6-desaturase / Fatty acid desaturase 2 (fads2) | Lipid metabolism | NM_001123575 | GGGATTTAATCCATCGCATATTAACT | CGTCACAACAAAATACAGCATCTG | 87 | 2.02 |
| *ptgs2* | Prostaglandin-endoperoxide synthase 2 / Cyclooxygenase 2 | Lipid metabolism | AY848944 | ACAGCCCCCCGACTTACAAT | GGTGTAGGGCAGTCCTTTGG | 112 | 2.04 |
| *srebf2* | Sterol regulatory element binding transcription factor 2 | Lipid metabolism | >Contig2187_Atlantic_salmon | CTGTCTGGCAACTGGCTCAA | CTCCCCATTGCTGCTGCTT | 105 | 2.12 |
| *scd* | Stearoyl CoA desaturase | Lipid metabolism | XM_014155112 | GACGACGCGTTCAATTGTCA | CGTGCCCAGTGTTGAGATGA | 122 | 1.97 |
| *cat* | Catalase | Oxidative stress | BG935638 | CCGACCGTCCGTAAATGCTA | GCTTTTCAGATAGGCTCTTCATGTAA | 141 | 2.05 |
|  |  |  |  |  |  |  |  |
| *eef1a2* | Eukaryotic translation elongation factor 1 alpha 2 | Reference gene | BG933853 | TGCCCCTCCAGGATGTCTAC | CAGTACCTGTGGGCCGTG | 57 | 2.00 |
| *actb* | Beta-actin | Reference gene | BG933897 | CCAAAGCCAACAGGGAGAAG | AGGGACAACACTGCCTGGAT | 91 | 1.94 |
|  |  |  |  |  |  |  |  |
|  |  |  |  |  |  |  |  |

**Table S2.** Results for PLS regression model for genes. Chlorpyrifos-methyl (CPM), pirimiphos-methyl (PPM) and nonylphenol (NP).

|  |  |  |  |  |  |  |  |  |  |  |  |  |
| --- | --- | --- | --- | --- | --- | --- | --- | --- | --- | --- | --- | --- |
|  |  |  |  |  |  |  |  |  |  |  |  |  |
| **Gene symbol** | **Gene name** | **PLS model** | | |  | **Coefficients** | | |  | **Interaction** |  | **Response** |
|  |  | **R^2^** | **Q^2^** | **Regression** |  | **PPM** | **CPM** | **NP** |  | **PPM+CPM** |  |  |
|  |  |  |  |  |  |  |  |  |  |  |  |  |
|  |  |  |  |  |  |  |  |  |  |  |  |  |
| *d5d* | Delta-5-desaturase (*fads1*) | 0.23 | 0.09 | 0.03 |  |  |  | 0.01 |  | 0.036 |  | Synergism and antagonism |
| *d6d* | Delta-6-desaturase (*fads2*) | 0.47 | 0.22 | 0 |  |  |  | 0 |  | 0.0007 |  | Antagonism and synergism |
| *scd* | Stearoyl-CoA desaturase | 0.45 | 0.2 | 0 |  |  |  | 0 |  | 0.011 |  | Antagonism and synergism |
| *srebf2* | Sterol regulatory element binding transcription factor 2 | 0.33 | 0,20 | 0.003 |  |  |  | 0 |  | n/a |  | No combined effect |
| *cat* | Catalase | 0.25 | 0.09 | 0.017 |  |  |  | 0.01 |  | 0.042 |  | Synergism and antagonism |
| *esr1* | Estrogen receptor 1 | 0.25 | 0.13 | 0.019 |  |  |  | 0.01 |  | 0.022 |  | Antagonism |
| *vtg* | Vitellogenin | 0.36 | 0.15 | 0.001 |  |  |  | 0 |  | 0.0023 |  | Antagonism and synergism |
| *cyp1a* | Cytochrome P450 family 1 subfamily A | 0.27 | 0.16 | 0.011 |  |  |  | 0 |  | n/a |  | No combined effect |
| *ugt1a* | UDP glucuronosyltransferase family 1 member A | 0.29 | 0.09 | 0.007 |  |  |  | 0.01 |  | 0.007 |  | Synergism and antagonism |
|  |  |  |  |  |  |  |  |  |  |  |  |  |
|  |  |  |  |  |  |  |  |  |  |  |  |  |
| R^2^=multiple correlation coefficient; Q^2^= cross-validated R^2^ and an estimate of the predictive ability of the model. N/a = not applicable. | | | | | | | | | |  |  |  |

**Table S3.** Results for PLS regression model for lipid species. Chlorpyrifos-methyl (CPM), pirimiphos-methyl (PPM) and nonylphenol (NP).

|  |  |  |  |  |  |  |  |
| --- | --- | --- | --- | --- | --- | --- | --- |
|  |  |  |  |  |  |  |  |
| **Lipid species** | **PLS model** | | | **Coefficients** | | | **Combined effect** |
|  | **R^2^** | **Q^2^** | **Regression** | **PPM** | **CPM** | **NP** |  |
|  |  |  |  |  |  |  |  |
|  |  |  |  |  |  |  |  |
| FFA(12:0) | 0.4360 | 0.1950 | 0.0240 |  |  | 0.0456 |  |
| FFA(20:0) | 0.4280 | 0.2310 | 0.0270 |  | 0.0105 |  |  |
| FFA(22:5) | 0.5510 | 0.3790 | 0.0040 |  | 0.0030 | 0.0215 | Yes |
| FFA(22:6) | 0.5930 | 0.3230 | 0.0020 |  | 0.0009 |  |  |
| LPC(18:0) | 0.8270 | 0.6880 | 0.0000 | 0.0015 | 0.0013 | 3,98162e-006 | Yes |
| PC(12:0/18:1) | 0.6360 | 0.4660 | 0.0010 |  |  | 9,19666e-005 |  |
| PC(18:0/20:4) | 0.3890 | 0.2650 | 0.0440 |  | 0.0088 |  |  |
| PE(18:1/20:5) | 0.5330 | 0.2040 | 0.0060 |  |  | 0.0007 |  |
| PE(18:1/22:6) | 0.6190 | 0.3710 | 0.0010 |  |  | 0.0001 |  |
| PE(18:2/22:6) | 0.4880 | 0.1900 | 0.0120 |  |  | 0.0015 |  |
| TAG50:1-FA16:0 | 0.3920 | 0.1390 | 0.0420 |  |  | 0.0441 |  |
| TAG52:1-FA16:0 | 0.424 | 0.1700 | 0.0280 | 0.0257 | 0.0475 |  | Yes |
| TAG52.:1-FA18:0 | 0.4330 | 0.1890 | 0.0250 | 0.0216 | 0.0403 |  | Yes |
| TAG54:1-FA18:0 | 0.3970 | 0.1610 | 0.0400 | 0.0231 |  |  |  |
|  |  |  |  |  |  |  |  |
|  |  |  |  |  |  |  |  |


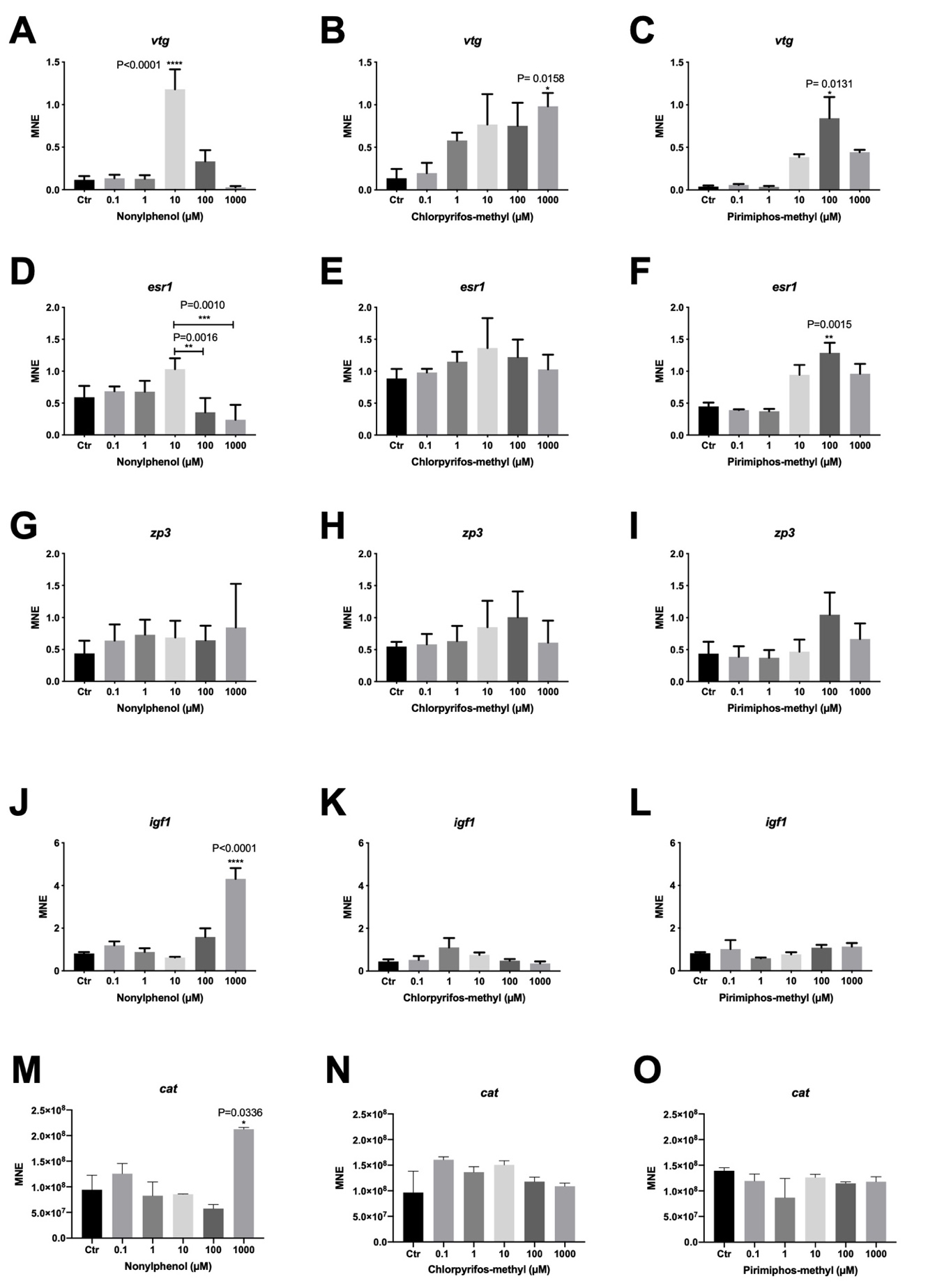


**Figure S1.** Dose-response relationships of genes associated with endocrine disruption, growth and oxidative stress in Atlantic salmon hepatocytes exposed to nonylphenol, chlorpyrifos-methyl and pirimiphos-methyl for 48 h. MNE=mean normalized expression. Asterisk’s denote significance based on one-way ANOVA analyses, with Holm Sidak’s posthoc test. * p < 0.05 (exact p-values given in figure), ** p < 0.01, *** p < 0.001, **** p < 0.0001.


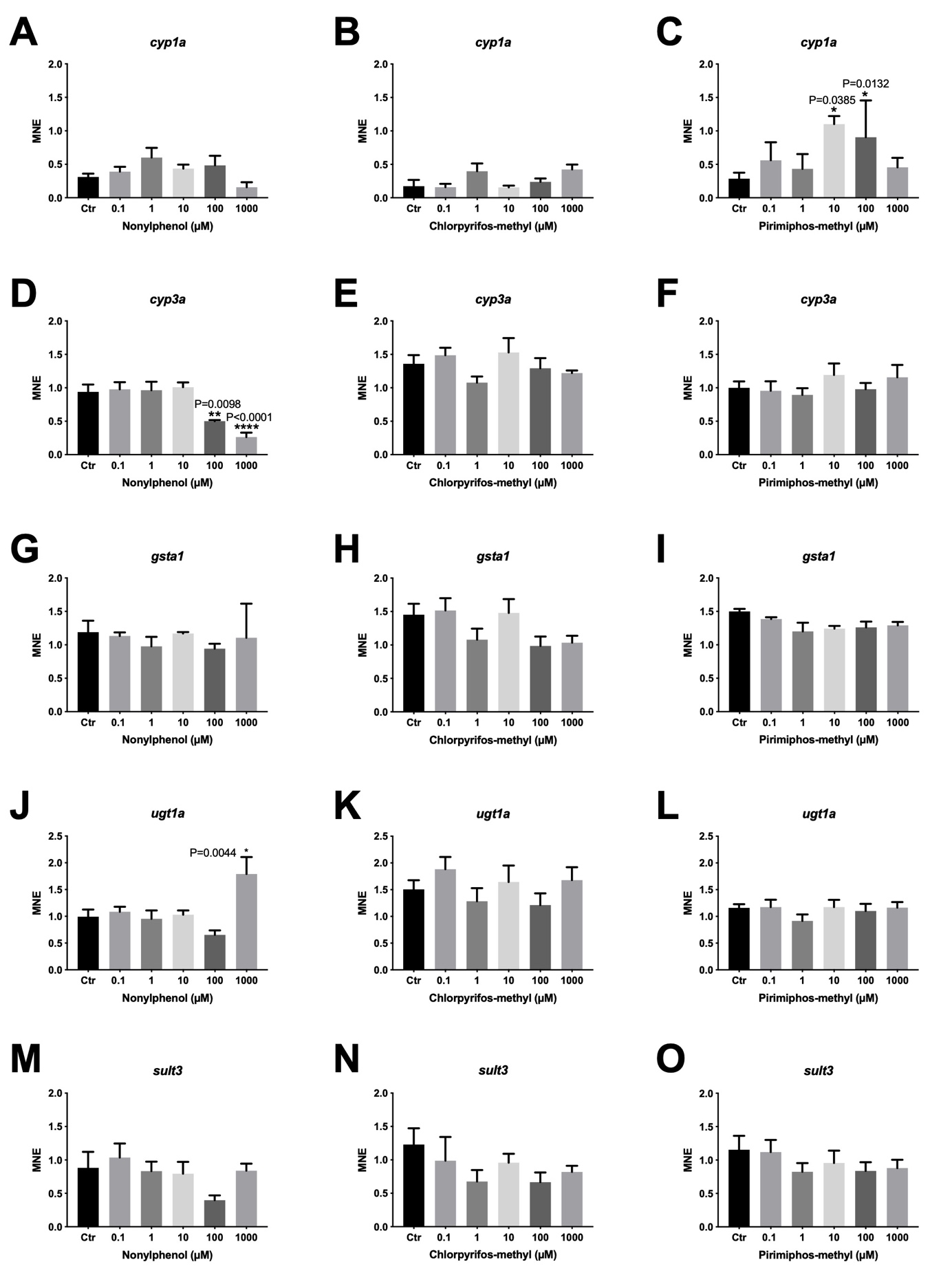


**Figure S2.** Dose-response relationships of genes associated with detoxification in Atlantic salmon hepatocytes exposed to nonylphenol, chlorpyrifos-methyl and pirimiphos-methyl for 48 h. MNE=mean normalized expression. Asterisk’s denote significance based on one-way ANOVA analyses, with Holm Sidak’s posthoc test. * p < 0.05 (exact p-values given in figure), ** p < 0.01, *** p < 0.001, **** p < 0.0001.


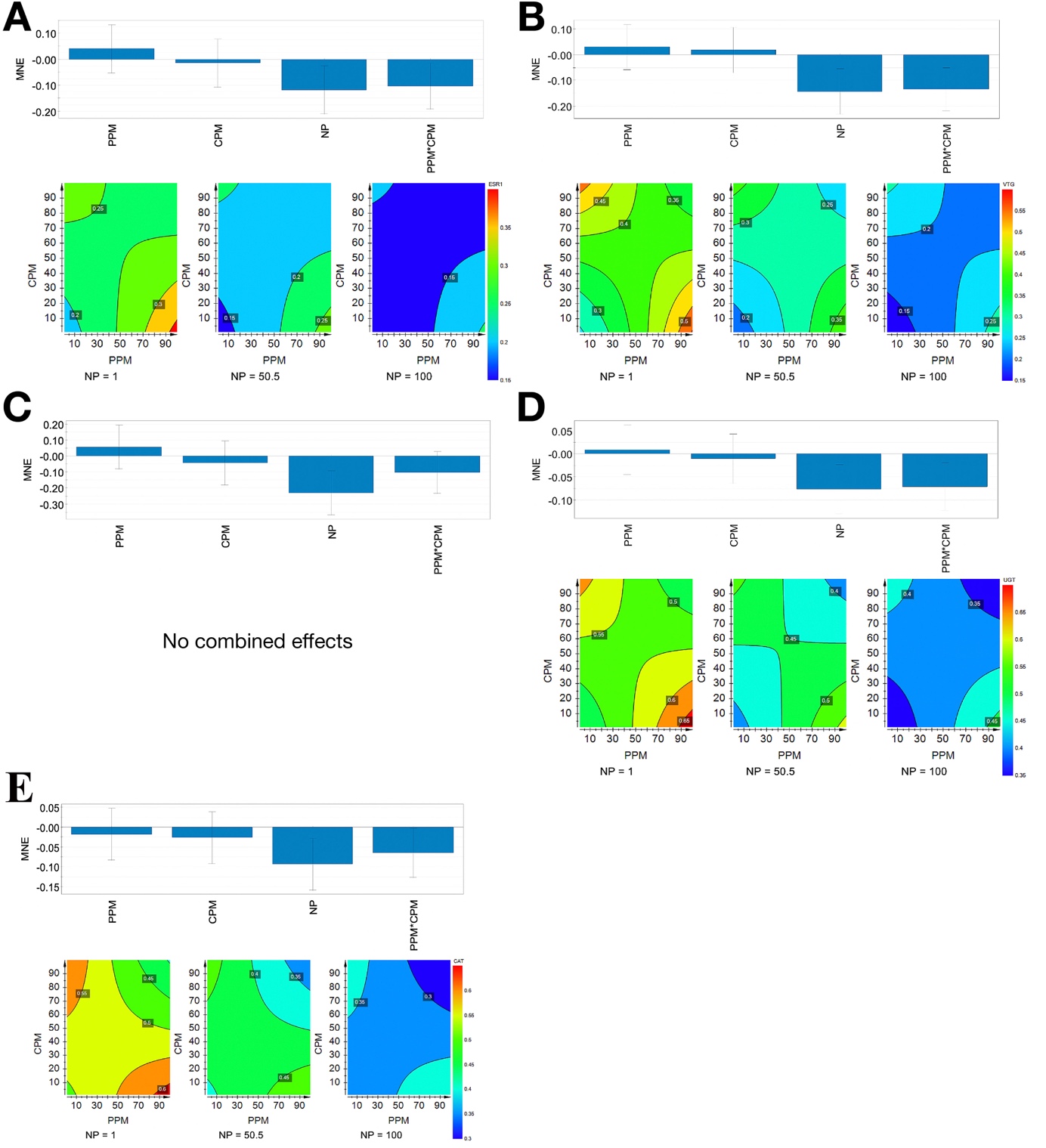


**Figure S3.** PLS models for endocrine disruption and detoxification marker genes. A) *esr1*, B) *vtg*, C) *cyp1a*, D) *ugt1a* and E) *cat*
